# Application of PET imaging delta radiomics for predicting progression-free survival in rare high-grade glioma

**DOI:** 10.1038/s41598-024-53693-x

**Published:** 2024-02-08

**Authors:** Shamimeh Ahrari, Timothée Zaragori, Adeline Zinsz, Julien Oster, Laetitia Imbert, Antoine Verger

**Affiliations:** 1 Imagerie Adaptative Diagnostique et Interventionnelle, Institut National de la Santé et de la Recherche Médicale U1254, Université de Lorraine, 54000 Nancy, France; 2 Nancyclotep Imaging Platform, Université de Lorraine, 54000 Nancy, France; 3 Department of Nuclear Medicine, Centre Hospitalier Régional Universitaire de Nancy, 54000 Nancy, France

**Keywords:** Image processing, Machine learning, Oncology, Tumour heterogeneity

## Abstract

This study assesses the feasibility of using a sample-efficient model to investigate radiomics changes over time for predicting progression-free survival in rare diseases. Eighteen high-grade glioma patients underwent two L-3,4-dihydroxy-6-[^18^F]-fluoro-phenylalanine positron emission tomography (PET) dynamic scans: the first during treatment and the second at temozolomide chemotherapy discontinuation. Radiomics features from static/dynamic parametric images, alongside conventional features, were extracted. After excluding highly correlated features, 16 different models were trained by combining various feature selection methods and time-to-event survival algorithms. Performance was assessed using cross-validation. To evaluate model robustness, an additional dataset including 35 patients with a single PET scan at therapy discontinuation was used. Model performance was compared with a strategy extracting informative features from the set of 35 patients and applying them to the 18 patients with 2 PET scans. Delta-absolute radiomics achieved the highest performance when the pipeline was directly applied to the 18-patient subset (support vector machine (SVM) and recursive feature elimination (RFE): C-index = 0.783 [0.744–0.818]). This result remained consistent when transferring informative features from 35 patients (SVM + RFE: C-index = 0.751 [0.716–0.784], p = 0.06). In addition, it significantly outperformed delta-absolute conventional (C-index = 0.584 [0.548–0.620], p < 0.001) and single-time-point radiomics features (C-index = 0.546 [0.512–0.580], p < 0.001), highlighting the considerable potential of delta radiomics in rare cancer cohorts.

## Introduction

Radiomics analysis of positron emission tomography (PET) imaging is of great interest, given its ability to characterize tumor heterogeneity and its prognostic value^1^. In neuro-oncology, radiomics studies have primarily focused on amino-acid PET radiotracers for glioma grading at the initial diagnosis^2,3^ and for predicting the presence of key molecular characteristics^4–9^ in accordance with the latest World Health Organization (WHO) classifications^10^. The potential benefits of radiomics for prognosis^11–13^, detecting recurrences^14–16^, and identifying early progression after glioma chemoradiation^17^ have also been evaluated. More specifically, studies have examined radiomics derived from both static and dynamic acquisitions at the initial diagnosis^18,19^, with the aim of assessing prognosis^20^ or identifying glioma recurrences^21^. Neuro-oncological applications of radiomics face a unique challenge, due to the relatively low occurrence of brain tumors worldwide, and therefore require the use of dedicated modeling workflows and/or data augmentation techniques^22,23^. Although gliomas account for almost 80% of newly diagnosed malignant primary brain tumors and the vast majority are high-grade glioblastomas^24^, gliomas only represent 2% of all adult cancers. Indeed, the worldwide age-standardized annual incidence of primary malignant brain tumors is approximately 3 per 100,000^25^. Despite the standard treatment, which involves surgical resection and adjuvant chemoradiation therapy^26,27^, our understanding of the prognosis for these tumors remains limited. The use of amino-acid PET radiotracers^28^, specifically L-3,4-dihydroxy-6-[^18^F]-fluoro-phenylalanine (^18^F-FDOPA), in conjunction with magnetic resonance imaging (MRI), is currently recommended^29^ as a non-invasive diagnostic approach for assessing glioma progression^30–32^. The application of amino-acid PET imaging by integrating dynamic^33^ and radiomics^34^ analyses, has had a significant impact in the field of neuro-oncology and has improved the diagnostic performance.

The traditional radiomics model utilizes single-time-point medical images to evaluate or predict patient outcomes, disregarding changes in tumor characteristics during treatment or influenced by external factors such as chemotherapy or radiotherapy. An alternative approach, known as delta radiomics, introduces a time component by extracting quantitative features from images acquired at different treatment and follow-up time points. The delta radiomics concept, which employs the change in radiomic features during or after treatment to guide clinical decisions, may potentially be more suitable for evaluating tumor response to treatment. Using machine-learning algorithms, delta radiomics analysis has shown a superior capacity to predict clinical outcomes compared to single-time-point radiomics features. Although a number of studies have focused on the application of delta radiomics in glioma patients, these have been restricted to MRI imaging and classification tasks^35–37^. Moreover, changes in radiomic features derived from PET/computed tomography (CT) imaging have also been assessed to predict survival outcomes in non-rare glioma cancers such as non-small cell lung cancer^38,39^ or head and neck carcinoma^40^ with effectiveness over at least 45 patients.

To the best of our knowledge, no study has to date explored the potential of amino-acid PET-based delta radiomics on time-to-event survival data in a rare disease such as glioma. The aim of our current study is therefore to evaluate the feasibility of using a sample-efficient model investigating radiomics changes over time to predict progression-free survival (PFS) in high-grade glioma (HGG). The objectives of this paper also include assessing the robustness of the model and comparing the delta radiomics results to both delta conventional features and single-time-point radiomics features.

## Material and methods

### Patient cohort

The clinical trial consisted in the retrospective analysis of images and medical records with the inclusion of 18 HGG patients with two consecutive PET scans, the first one during treatment (${PET}_{0}$) and the second at the time of adjuvant temozolomide (TMZ) chemotherapy discontinuation (${PET}_{1}$), allowing to assess changes in extracted features over time. This population of 18 patients, was selected as a subset from a total of 53 patients who underwent dynamic ^18^F-FDOPA PET scans, after following a Stupp protocol, in the Nuclear Medicine Department of the Regional University Hospital (CHRU) of Nancy, between January 2018 and May 2022. Minimum follow-up was 1 year. The decision to treat with the Stupp protocol and initiate or discontinue adjuvant TMZ therapy was determined individually for each patient during the multidisciplinary neuro-oncology tumor board meetings. Tumors were classified as HGG based on the surgical sample or biopsies, in accordance with the WHO 2021 classification guidelines^10^. Following the RANO^41^ criteria, the clinical endpoint of PFS was defined as the time in months from the initial treatment to either the date of progression or censoring. Informed consent was obtained from all patients. The institutional ethics committee (Comité d'Éthique du CHRU de Nancy) approved the study, on 26 August 2020. The trial was registered at ClinicalTrials.gov (NCT04469244) and complied with the principles of the Helsinki declaration.

### Data acquisition

Prior to undergoing ^18^F-FDOPA PET scans, all patients fasted for a minimum of 4 h. Several patients received Carbidopa 1 h prior to the examination to enhance tracer uptake in the brain^42^. This institutional procedure was implemented from February 2018 to September 2020. PET acquisitions were performed with a digital PET/CT device (Vereos, Philips Healthcare^®^, Eindhoven, The Netherlands). Each patient initially underwent a CT scan, then, following the injection of 2MBq of ^18^F-FDOPA per kg of body weight, a 30-min dynamic PET acquisition was performed. Using an OSEM 3D algorithm, a static image was reconstructed based on the last 20 min of the acquisition (2 iterations, 10 subsets, 256 × 256 × 164 voxels of 1 × 1 × 1 mm^3^). Dynamic images were reconstructed with 30 frames, each lasting 1 min (3 iterations, 15 subsets, 128 × 128 × 82 voxels of 2 × 2 × 2 mm^3^). During this process, images were corrected for attenuation, dead time as well as random and scattered coincidences. Point-spread function corrections were only applied to static images^43^.

### Image pre-processing

An experienced nuclear physician (A.Z.) manually delineated, healthy brain, striatum, and tumor volumes of interest (VOIs) on static images with the LifeX software^44^ (lifexsoft.org). For patients who underwent 2 PET scans, different segmented VOIs from both ${PET}_{0}$ and ${PET}_{1}$ were considered. The healthy brain VOI was selected as a crescent-shaped region on three consecutive slices of the semi-oval center from the unaffected hemisphere^29^. Semi-automatic segmentation was performed using a 70% threshold of the maximum standardized uptake value (SUV_max_) for the striatum VOI, and a threshold of 1.6 times the mean SUV (SUV_mean_) of healthy brain for the tumor VOI, to yield the metabolic tumor volume (MTV)^29^. Dynamic images were registered on the CT to correct for patient movement. Considering our focus on the voxel-based analysis and the need to reduce the impact of noise on voxel time activity curves (TACs), dynamic images underwent a denoising process^45^. As recommended elsewhere^46^, the impact of Carbidopa on SUV measurements was reduced by normalizing static images to the SUV_mean_ of healthy brain, which provided static tumor-to-background-ratio (TBR) parametric images. For dynamic images, region/voxel-based TACs were initially fitted and subsequently normalized to the fitted mean brain TACs. Time-to-peak (TTP) values, representing the time interval between the start of acquisition and maximum uptake, were extracted. This procedure generated dynamic TTP parametric images for the voxel-based analysis.

### Radiomics analysis

#### Feature extraction

The feature extraction step applied two distinct approaches i.e., the region- and voxel-based analysis. For the region-based analysis, conventional features such as mean, maximum and peak of TBR, and tumor-to-striatum ratios were extracted from the tumor VOIs. We also obtained the MTV, region-based dynamic TTP, and calculated the slope of the linear regression for the data acquired between the 10th and 30th min^4^, which provided 9 conventional features. For the voxel-based analysis, we initially resampled the dynamic TTP parametric images with a linear interpolation, to achieve a common resolution of 1 × 1 × 1 mm^3^ with the static TBR parametric images, as described by the Image Biomarker Standardization Initiative^47^. First-order statistics, morphological, intensity histogram, and textural features were extracted using the pyradiomics package^48^.

The previously described in-house software^4^ was used to identify local intensity features (https://github.com/TimZaragori/Sklearn_NestedCV/blob/master/Radiomics_gliomas_article/local_intensity_features.py) that were not available in pyradiomics. Discretization of the parametric images was performed using a fixed bin width of 0.1 for the static TBR and 1 min for dynamic TTP parametric images, resulting in 199 extracted radiomics features (94 static TBR radiomics features, 94 dynamic TTP radiomics features and 11 morphological features).

#### Feature sets

Four methods were used to compute changes in radiomics and conventional features. These included delta-absolute (${F}_{{PET}_{1}}- {F}_{{PET}_{0}}$, with ΔAR and ΔAC defined as delta-absolute radiomics and conventional features, respectively) and delta-relative ($\left({F}_{{PET}_{1}}- {F}_{{PET}_{0}}\right)/{F}_{{PET}_{0}}$, where ΔRR and ΔRC represent delta-relative radiomics and conventional features, respectively).

In addition, we also evaluated weighted delta-absolute ($\left({F}_{{PET}_{1}}- {F}_{{PET}_{0}}\right)/\left({t}_{{PET}_{1}}- {t}_{{PET}_{0}}\right)$, by considering WΔAR and WΔAC for weighted delta-absolute radiomics and conventional features, respectively) and weighted delta-relative ($\left({F}_{{PET}_{1}}- {F}_{{PET}_{0}}\right)/{F}_{{PET}_{0}}\left({t}_{{PET}_{1}}- {t}_{{PET}_{0}}\right)$, with WΔRR and WΔRC referring to weighted delta-relative radiomics and conventional features, respectively), which normalized the delta features by the time interval between the two collected PET scans. The analysis also included the single-time-point feature (${F}_{{PET}_{1}}$, where STPR refers to the single-time-point radiomics feature). Here, ${F}_{{PET}_{0}}$, ${F}_{{PET}_{1}}$ represent the extracted features and ${t}_{{PET}_{0}}$ and ${t}_{{PET}_{1}}$ indicate the acquisition dates of the PET scans collected during treatment and after the last treatment sessions respectively.

### Feature selection methods and machine learning algorithms

To reduce the risk of overfitting in this small patient cohort, several steps were considered in the applied pipeline. For a given training and test set, all transformations and algorithms were initially fitted exclusively to the training set and then applied to the test set for output prediction. After the feature extraction, variables with zero variance were eliminated, while the other variables underwent Z-score normalization. To address the problem of highly correlated features, we used hierarchical clustering based on the absolute Spearman correlation coefficient as a similarity metric, with a threshold of 0.9 to form the clusters^49^. The selected feature was the medoid of each cluster, which represents the most centrally located feature in terms of maximizing the similarity to all other features within the same cluster. Due to the limited number of samples, where the algorithm is sensitive to small perturbations, the consensus clustering^50^ technique was implemented to enhance the stability and robustness of the clustering process. This involved performing the clustering on each of the 500 bootstrap samples extracted from each training fold, resulting in 500 clustering outcomes, each representing a different arrangement of features. The final clusters were then determined by comparing the cluster assignments using a co-association matrix, based on a 0.5 threshold. This matrix represents the frequency or probability of features being assigned to the same cluster across multiple clustering runs.

In order to obtain non-redundant features after the clustering step, feature selection was performed and we compared several approaches including univariate concordance index (C-index), mutual information (MI), LASSO Cox and recursive feature elimination (RFE) methods to identify the most informative features. For the filter-based feature selection methods, i.e. univariate C-index and MI, the process was repeated 500 times using bootstrap samples from each training fold with features ranked according to the score in each bootstrap sample and overall rank computed with the importance score^49^. To model time-to-event survival data, Cox proportional hazards (CoxPH), ElasticNet Cox, support vector machine (SVM), and gradient boosting with component-wise least squares as base learner (GB-Linear) algorithms^51^ were employed, resulting in a total of 16 different models being trained (4 feature selections multiplied by 4 regressors).

### Model training strategy

**Figure 1:**
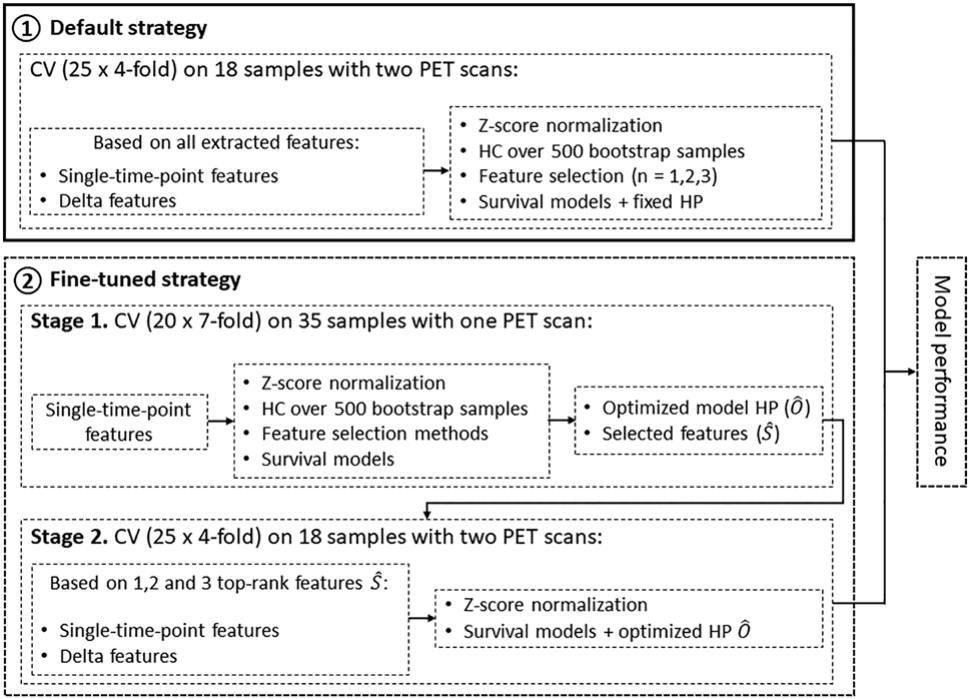
Flowchart describing the overall research pipeline. CV: cross-validation; PET: positron emission tomography; HC: hierarchical clustering; HP: hyperparameter.

Two different strategies (Fig. 1) were explored for predicting PFS based on feature changes using the described pipeline.

In the main strategy, referred to as the default strategy, the pipeline was directly applied to the subset of 18 samples with 2 PET scans. Due to the small number of samples, the model hyperparameters were fixed, without undergoing further optimization (Supplementary Table S1). In this strategy, the feature selection methods were restricted to select consecutively 1, 2, and 3 single-time-point and delta features for comparison purpose. Model performance was evaluated using a 4-fold cross-validation (CV) with 25 repetitions.

The second strategy, serving as a validation of the first, is termed the fine-tuned strategy and involves a two-stage process. Initially, optimized hyperparameters and informative features were obtained from a separate and larger sample set (stage 1) and subsequently transferred to a smaller cohort of interest (stage 2). Focusing on single-time-point features in stage 1, information was extracted from all the patient with a single PET scan only (n = 35). This information was then used to build the model for stage 2, specifically aimed at evaluating the delta features extracted from the 18 patients with 2 PET scans. During stage 1, a 7-fold CV (ensuring the presence of at least 5 samples in the validation set) was repeated 20 times. The model hyperparameters were optimized (Supplementary Table S1) through a Bayesian search using Gaussian processes (https://github.com/scikit-optimize/scikit-optimize) over 100 iterations, with a focus on maximizing the C-index. Feature selection methods were limited to selecting at most 3 single-time-point radiomics and conventional features. In stage 1, the importance of features was calculated for each model across all folds using SHapley Additive exPlanations^52^ (SHAP) values, and subsequently, single-time-point features were ranked based on their importance. In stage 2, the analysis of single-time-point and delta features focused on the previously optimized hyperparameters in stage 1 and compared models by consecutively inputting 1, 2, and 3 top-ranked features identified in stage 1. The final model performance was assessed during stage 2 through a repeated CV process (4 folds, 25 repetitions) on the population of 18 patients with 2 PET scans.

The results were finally compared to a CoxPH model applied to conventional features only, which served as the baseline model (Baseline). The CoxPH model was also used to evaluate both absolute and relative changes of TBR_max_ and TBR_mean_ for a univariate comparison.

### Model robustness evaluation

Firstly, to address the limited number of samples with 2 PET scans, a fine-tuned strategy was developed (Fig. 1), transferring information from a larger set (n = 35) with single-time-point features to assess delta features within the smaller set (n = 18), serving as a validation of the default strategy.

Secondly, the results of different models employed for the default strategy were compared in terms of performance.

Thirdly, the applied pipeline integrated the bootstrap-based consensus clustering technique to improve cluster stability in each fold. The adjusted rand index^53^ (ARI), used as a similarity measure between two clustering results, was computed between the clustering results of all possible CV fold pairs and presented as the average of these pairs. Cluster similarity was determined by a negative ARI for discordant clustering, an ARI close to 0 for random labeling, and an ARI close to 1 when the clustering results were identical. ARI was also used to assess the similarity between the clusters of the first fold, arbitrary considered as the reference fold, and those derived from subsequent folds. For each cluster of the first fold, the most analogous cluster and the corresponding medoid features were identified, allowing us to evaluate the stability of these medoid features throughout CV folds.

Lastly, feature importance was calculated through SHAP values to assess whether the same features were selected across various models of the default strategy.

### Statistical analysis

Categorical variables are expressed as counts and percentages and continuous variables as medians with interquartile ranges (IQR). The model performance on the test set was evaluated using the C-index, which assesses the probability that the sample with the highest predicted risk experiences an event before the sample with the lowest predicted risk among all the possible pairs. The integrated time-dependent area under the curve (iAUC) was calculated as a complementary metric to address potential limitations of the C-index. The iAUC estimates how effectively a predictive model can differentiate individuals who will experience an event within a specific time period from those who will not. The final result was obtained from one thousand bootstrap iterations of the CV performance distribution and summarized as the mean and standard error values with a 95% confidence interval (CI). To evaluate whether delta radiomics are superior to both delta conventional features and single-time-point radiomics features, one-sided Mann–Whitney U tests were conducted on the C-indices. To account for multiple comparisons, the p-values were corrected using the Benjamini-Hochberg^54^ method. A p-value < 0.05 was considered statistically significant. All analyses were performed in Python 3.8, using the scikit-survival^51^ library. Important features were identified using SHAP values for both the default strategy model and the second stage of the fine-tuned strategy.

## Results

### Patient characteristics

Among the 53 HGG patients identified, 18 patients with the following characteristics had undergone 2 PET scans (Supplementary Table S2). The median age was 62 years (IQR, 45–69) and the patients included 6 (33%) women. The median PFS was 11 months (IQR, 8–21), with a total of 16 (89%) patients relapsing. Carbidopa premedication was administered to 5 (28%) patients prior to ^18^F-FDOPA PET acquisition. The patients underwent either surgery (78%) or biopsy (22%) with tumors classified at the initial diagnosis according to the WHO 2021 glioma classification^10^. There were 3 (17%) IDH-mutant and 1p/19q non-codeleted astrocytomas (67% grade III and 33% grade IV) and 15 (83%) IDH-wildtype glioblastomas.

### Survival analysis

**Figure 2:**
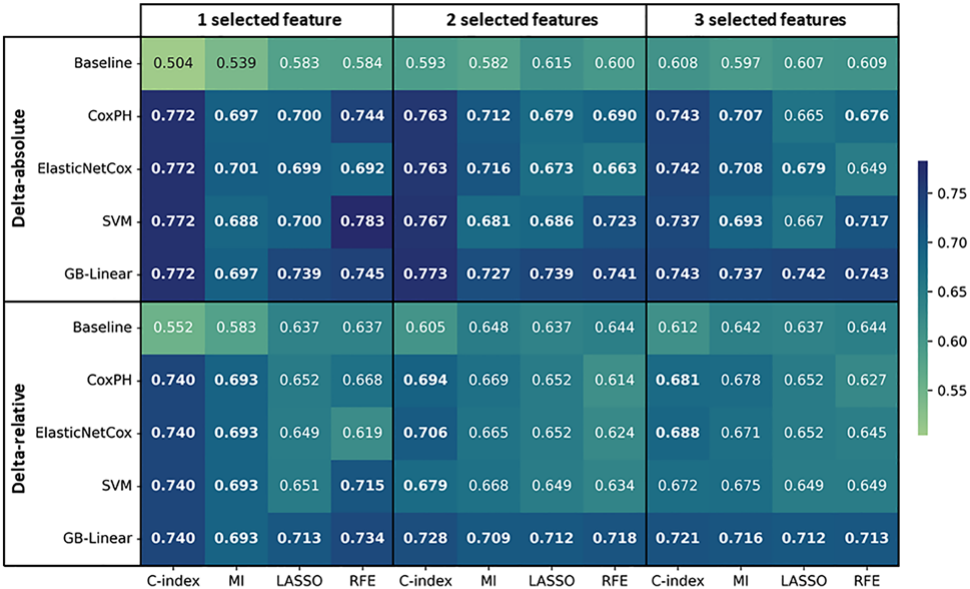
Heatmap representing the C-indices of the default strategy based on the machine-learning algorithms (y-axis) and feature selection methods (x-axis) of the test set. The bold values indicate a significant p-value when comparing the radiomics model with its respective Baseline. GB: gradient boosting; SVM: support vector machine; MI: mutual information; RFE: recursive feature elimination.

Results obtained by selecting 1, 2, and 3 features were largely consistent (Fig. 2). The highest prediction performance was obtained with the SVM model in combination with RFE for delta-absolute radiomics (ΔAR: C-index = 0.783 [0.744, 0.818]) and with C-index feature selection for delta-relative radiomics (ΔRR: C-index = 0.740 [0.700, 0.778]), both based on a single selected feature (Fig. 2). Interestingly, GLCM Information Correlation 2 from dynamic TTP parametric images emerged as the most important radiomics feature in both models. The previously mentioned SVM + RFE model for delta-absolute and the SVM + C-index model for delta-relative features demonstrated significantly better performance for delta radiomics compared to single-time-point radiomics (Table 1), highlighting the positive influence of delta radiomics features on model decision-making (STPR of SVM + RFE: C-index = 0.546 [0.512, 0.580], p < 0.001 and STPR of SVM + C-index: C-index = 0.555 [0.514, 0.594], p < 0.001). These models also displayed a significantly better result for the delta radiomics compared to the delta conventional features (ΔAC of SVM + RFE: C-index = 0.584 [0.548, 0.620], p < 0.001 and ΔRC of SVM + C-index: C-index = 0.552 [0.513, 0.589], p < 0.001, when comparing the results of delta-absolute/relative radiomics features with their corresponding pairs of conventional features). Weighted delta radiomics results (WΔAR of SVM + RFE: C-index = 0.721 [0.682, 0.759] and WΔRR of SVM + C-index: C-index = 0.719 [0.682, 0.757], Supplementary Table S3) indicated a similar trend, with a better performance compared to single-time-point radiomics (p < 0.001 for both WΔAR and WΔRR) and weighted delta conventional features (WΔAC of SVM + RFE: C-index = 0.533 [0.492, 0.577], p < 0.001 and WΔRC of SVM + C-index: C-index = 0.451 [0.408, 0.495], p < 0.001).

**Table 1:** Performance of the default strategy based on one selected feature, using the SVM + RFE model for delta-absolute and the SVM + C-index model for delta-relative features.

Features/metrics	Training		Test	
	iAUC	C-index	iAUC	C-index
Univariate CoxPH				
ΔATBR_max_	0.721 ± 0.004 [0.714, 0.729]	0.656 ± 0.004 [0.650, 0.663]	0.710 ± 0.019 [0.672, 0.744]	0.668 ± 0.016 [0.635, 0.696]
ΔRTBR_max_	0.721 ± 0.004 [0.713, 0.729]	0.665 ± 0.004 [0.657, 0.673]	0.709 ± 0.021 [0.667, 0.746]	0.672 ± 0.017 [0.636, 0.704]
ΔATBR_mean_	0.666 ± 0.004 [0.658, 0.674]	0.625 ± 0.003 [0.620, 0.631]	0.681 ± 0.019 [0.643, 0.716]	0.650 ± 0.015 [0.620, 0.680]
ΔRTBR_mean_	0.680 ± 0.004 [0.672, 0.688]	0.632 ± 0.004 [0.625, 0.638]	0.694 ± 0.019 [0.658, 0.729]	0.657 ± 0.015 [0.627, 0.685]
SVM + RFE				
STPR	0.802 ± 0.005 [0.791, 0.811]	0.719 ± 0.004 [0.711, 0.726]	0.554 ± 0.023 [0.513, 0.597]	0.546 ± 0.018 [0.512, 0.580]
ΔAC	0.711 ± 0.006 [0.699, 0.723]	0.666 ± 0.004 [0.658, 0.674]	0.599 ± 0.023 [0.556, 0.642]	0.584 ± 0.019 [0.548, 0.620]
ΔAR	0.901 ± 0.003 [0.894, 0.908]	0.826 ± 0.004 [0.818, 0.834)]	0.815 ± 0.018 [0.775, 0.850]	0.783 ± 0.018*^¥#§^ [0.744, 0.818]
SVM + C-index				
STPR	0.793 ± 0.006 [0.779, 0.806]	0.717 ± 0.003 [0.710, 0.724]	0.559 ± 0.024 [0.510, 0.606]	0.555 ± 0.020 [0.514, 0.594]
ΔRC	0.805 ± 0.006 [0.793, 0.818]	0.738 ± 0.005 [0.728, 0.749]	0.542 ± 0.024 [0.494, 0.586]	0.552 ± 0.020 [0.513, 0.589]
ΔRR	0.904 ± 0.003 [0.897, 0.911]	0.832 ± 0.004 [0.825, 0.840]	0.775 ± 0.021 [0.732, 0.816]	0.740 ± 0.020*^¥#§^ [0.700, 0.778]

*p-value significant for delta radiomics compared to delta conventional features; ^¥^p-value significant for delta radiomics compared to single-time-point radiomics; ^#^p-value significant for delta radiomics compared to univariate delta TBR_max_; ^§^p-value significant for delta radiomics compared to univariate delta TBR_mean_. iAUC: integrated time-dependent area under the curve; ΔATBR_max_: delta-absolute of maximum tumor-to-brain ratio; ΔRTBR_max_: delta-relative of maximum tumor-to-brain ratio; ΔATBR_mean_: delta-absolute of mean tumor-to-brain ratio; ΔRTBR_mean_: delta-relative of mean tumor-to-brain ratio; STPR: single-time-point radiomics; ΔAC: delta-absolute conventional; ΔAR: delta-absolute radiomics; ΔRC: delta-relative conventional; ΔRR: delta-relative radiomics; SVM: support vector machine; RFE: recursive feature elimination.

### Model robustness evaluation

**Figure 3:**
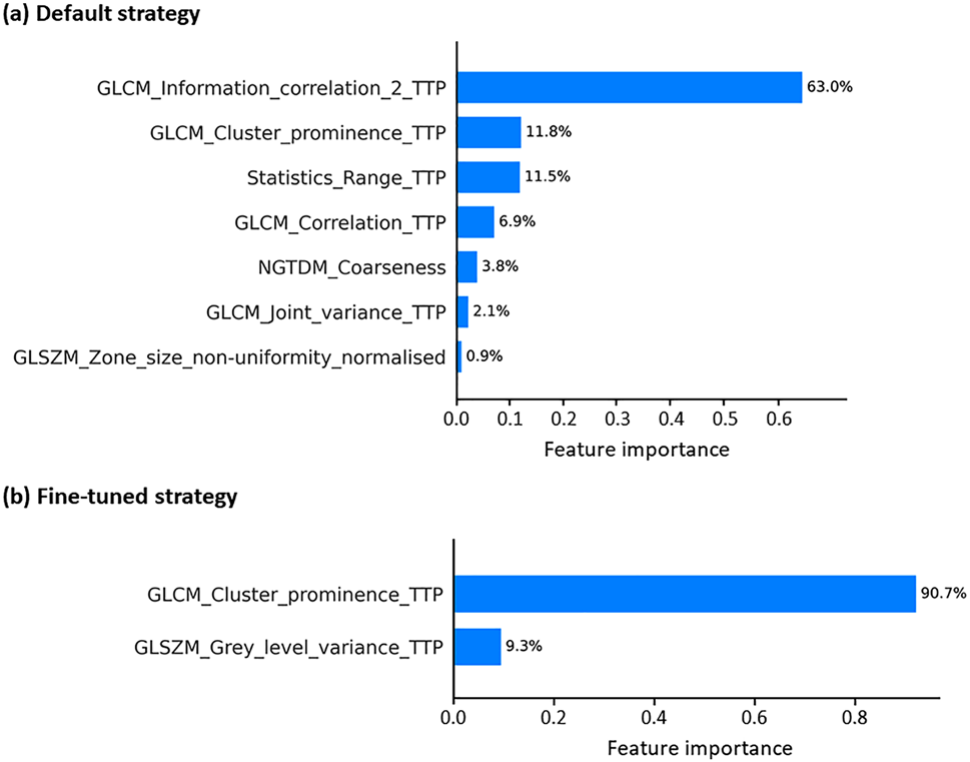
SHAP values indicate the feature importance in (**a**) the default strategy based on a single selected delta-absolute radiomics, and in (**b**) the second stage of the fine-tuned strategy based on two selected delta-absolute radiomics employing the SVM + RFE model in both strategies. The presence of TTP at the end of a feature name indicates that the corresponding feature is extracted from dynamic TTP parametric images.

Firstly, the fine-tuned strategy demonstrated optimal performance using the same combination SVM + RFE model as those used with the default strategy but with 2 selected features (Supplementary Fig. S1), revealing the delta-absolute and relative GLCM Cluster Prominence from dynamic TTP parametric image as the most informative radiomics features in stage 2 (ΔAR: C-index = 0.751 [0.716, 0.784] and ΔRR: C-index = 0.779 [0.742, 0.812], Table 2). Aligned with the default strategy, delta radiomics in the fine-tuned strategy also performed better than those obtained with single-time-point radiomics (p < 0.001 for both delta-absolute/relative radiomics) and delta conventional features (p < 0.001 for both delta-absolute/relative radiomics). Results from weighted delta radiomics reinforced this observation (see Supplementary Table S4), outperforming both single-time-point radiomics (p < 0.001 for both weighted delta-absolute/relative radiomics) and weighted delta conventional features (p = 0.006 for weighted delta-absolute, and p = 0.007 for weighted delta-relative radiomics). In both strategies, delta radiomics derived from the dynamic TTP parametric image and notably belonging to the same second-order radiomics family (GLCM) emerged as the most informative attribute (Fig. 3).

**Table 2:** Performance of the fine-tuned strategy based on two selected features, using the SVM + RFE model for delta-absolute/relative features.

Features/metrics	Training		Test	
	iAUC	C-index	iAUC	C-index
Univariate CoxPH				
ΔATBR_max_	0.721 ± 0.004 [0.714, 0.729]	0.656 ± 0.004 [0.650, 0.663]	0.710 ± 0.019 [0.672, 0.744]	0.668 ± 0.016 [0.635, 0.696]
ΔRTBR_max_	0.721 ± 0.004 [0.713, 0.729]	0.665 ± 0.004 [0.657, 0.673]	0.709 ± 0.021 [0.667, 0.746]	0.672 ± 0.017 [0.636, 0.704]
ΔATBR_mean_	0.666 ± 0.004 [0.658, 0.674]	0.625 ± 0.003 [0.620, 0.631]	0.681 ± 0.019 [0.643, 0.716]	0.650 ± 0.015 [0.620, 0.680]
ΔRTBR_mean_	0.680 ± 0.004 [0.672, 0.688]	0.632 ± 0.004 [0.625, 0.638]	0.694 ± 0.019 [0.658, 0.729]	0.657 ± 0.015 [0.627, 0.685]
SVM + RFE				
STPR	0.628 ± 0.005 [0.617, 0.639]	0.612 ± 0.004 [0.603, 0.620]	0.633 ± 0.023 [0.589, 0.677]	0.607 ± 0.019 [0.570, 0.643]
ΔAC	0.709 ± 0.004 [0.701, 0.717]	0.656 ± 0.004 [0.648, 0.663]	0.701 ± 0.020 [0.659, 0.737]	0.659 ± 0.017 [0.623, 0.689]
ΔAR	0.835 ± 0.002 [0.831, 0.840]	0.762 ± 0.003 [0.757, 0.767]	0.790 ± 0.018 [0.751, 0.825]	0.751 ± 0.016*^¥#§^ [0.716, 0.784]
ΔRC	0.719 ± 0.004 [0.710, 0.727]	0.656 ± 0.004 [0.648, 0.663]	0.703 ± 0.020 [0.660, 0.738]	0.664 ± 0.017 [0.627, 0.694]
ΔRR	0.817 ± 0.003 [0.810, 0.823]	0.752 ± 0.004 [0.745, 0.759]	0.819 ± 0.017 [0.779, 0.852]	0.779 ± 0.017*^¥#§^ [0.742, 0.812]

*p-value significant for delta radiomics compared to delta conventional features; ^¥^p-value significant for delta radiomics compared to single-time-point radiomics; ^#^p-value significant for delta radiomics compared to univariate delta TBR_max_; ^§^p-value significant for delta radiomics compared to univariate delta TBR_mean_. iAUC: integrated time-dependent area under the curve; ΔATBR_max_: delta-absolute of maximum tumor-to-brain ratio; ΔRTBR_max_: delta-relative of maximum tumor-to-brain ratio; ΔATBR_mean_: delta-absolute of mean tumor-to-brain ratio; ΔRTBR_mean_: delta-relative of mean tumor-to-brain ratio; STPR: single-time-point radiomics; ΔAC: delta-absolute conventional; ΔAR: delta-absolute radiomics; ΔRC: delta-relative conventional; ΔRR: delta-relative radiomics.

**Figure 4:**
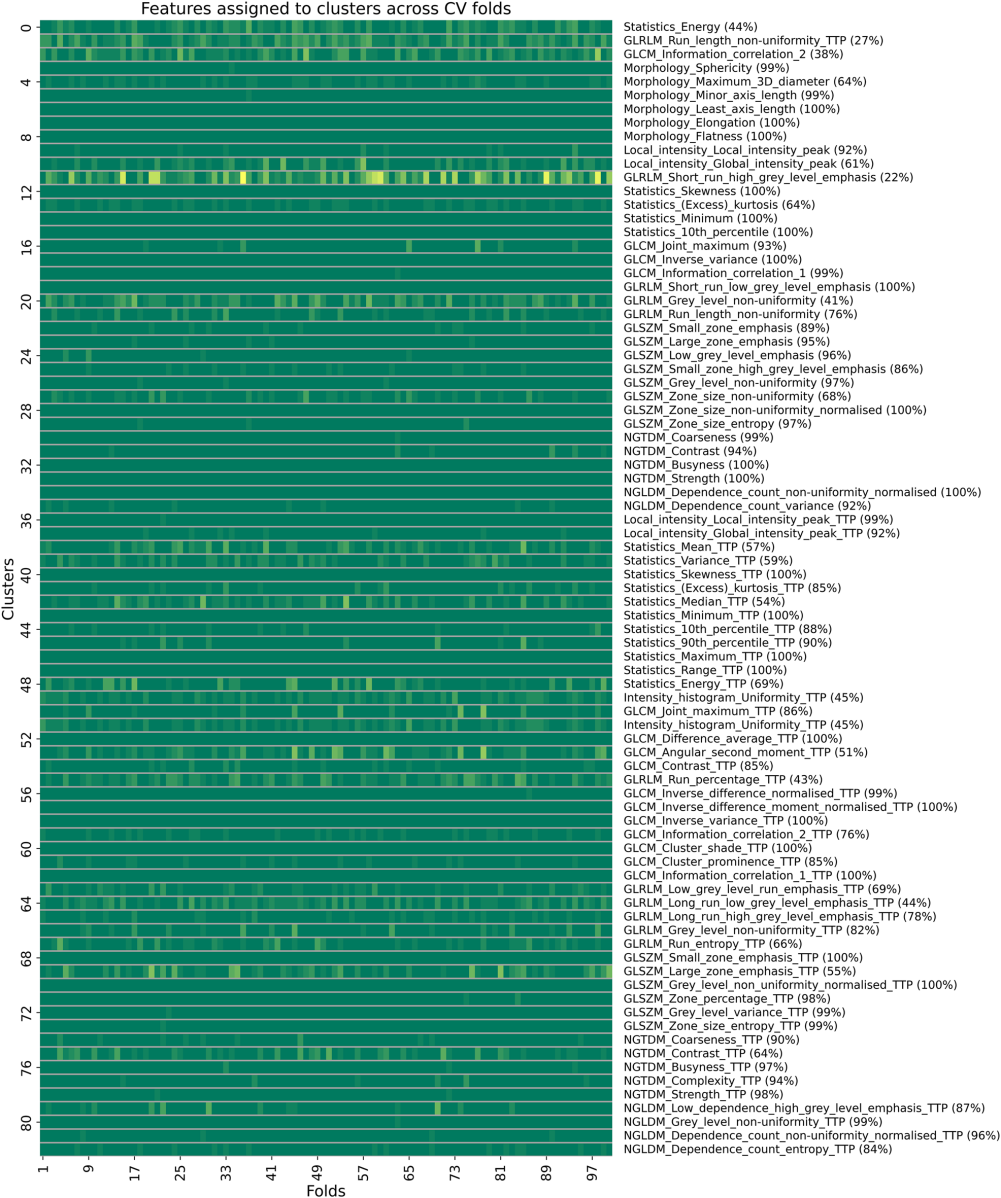
The consistency of medoid features assigned to clusters across CV folds of the default strategy, based on a single selected feature and using the SVM + RFE model. The right side of the figure exhibits the medoid feature observed in the majority of folds, along with its corresponding percentage. The presence of TTP at the end of a feature name indicates that the corresponding feature is extracted from dynamic TTP parametric images. CV: cross-validation; GLRLM: gray level run length matrix; GLCM: gray level co-occurrence matrix; GLSZM: gray level size zone matrix; NGTDM: neighboring gray tone difference matrix; NGLDM: neighboring gray level dependence matrix.

**Figure 5:**
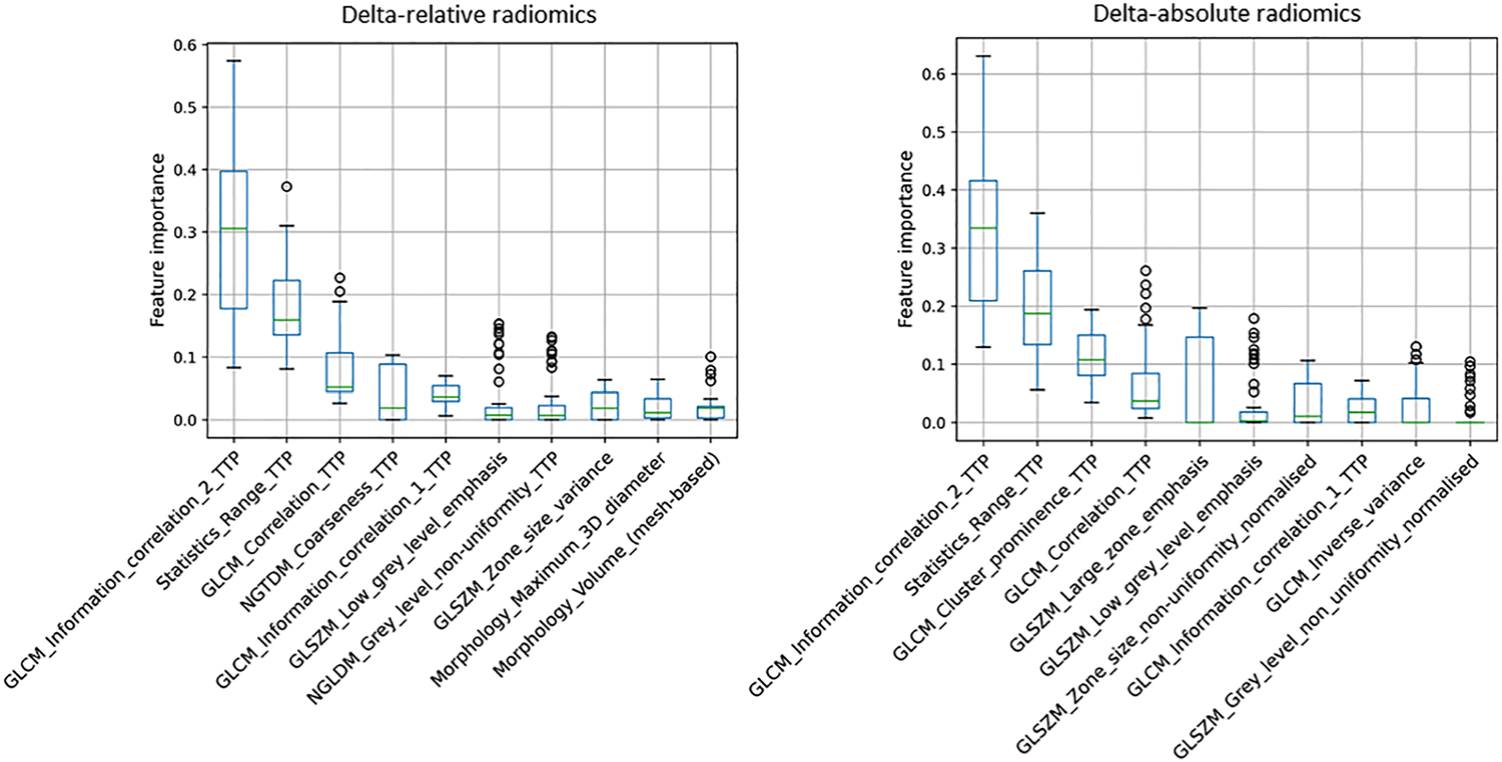
SHAP value distributions for the top 10 ranked features across all 16 model combinations based on 1, 2, and 3 selected features in each CV fold within the default strategy. The presence of TTP at the end of a feature name indicates that the corresponding feature is extracted from dynamic TTP parametric images. CV: cross-validation; GLCM: gray level co-occurrence matrix; GLSZM: gray level size zone matrix; NGTDM: neighboring gray tone difference matrix; NGLDM: neighboring gray level dependence matrix.

Secondly, in the default strategy, the methods employed to calculate changes in radiomics features did not significantly influence the results (the C-index range of 0.688 to 0.783 for delta-absolute and 0.619 to 0.740 for delta-relative, each based on a single selected feature), indicating a reliable extraction of relevant information from the input features by the model (Fig. 1). Similarly, the majority of radiomics models in the default strategy outperformed their conventional pairs (Fig. 1). Implementing bootstrap-based consensus clustering efficiently reduced the dimension of highly correlated features, resulting in a mean ARI value of 0.59 across CV folds for the SVM + RFE and a single selected feature within this strategy. Despite moderate cluster stability (ARI = 0.59), 70% of the medoid features assigned to the clusters were consistently observed in at least 80% of CV folds (Fig. 4). Furthermore, subsequent feature selection demonstrated consistency, with the GLCM Information correlation 2 extracted from dynamic TTP parametric images provided as the highest rank feature in terms of importance across all model combinations (Fig. 5).

## Discussion

This study evaluated the prognostic value of delta radiomics features extracted from ^18^F-FDOPA PET scans acquired during HGG treatment and after the final treatment session and how these correlated with time-to-event survival data. Our results demonstrate that delta radiomics can be effectively performed and evaluated in rare cancer cohorts and that they outperform delta conventional features and single-time-point radiomics features. Notably, the delta radiomics model applied to rare cohorts in this study has demonstrated some criteria of robustness.

To the best of our knowledge, this study is the first to investigate the potential of PET-based radiomics changes over time to predict PFS in a relatively small cohort, typical of rare disease patient cohorts such as glioma. The aim of this methodological approach was to assess whether delta radiomics features were amenable to small patient numbers and how were their performances compared to both delta conventional features and single-time-point radiomics features. In addition to extracting single-time-point features from the PET scan conducted after the final treatment session, we also considered the absolute and relative changes of the extracted features, as well as their normalized versions, taking into account the time intervals between the 2 PET scans. We developed a robust pipeline incorporating key steps to enhance the reliability of the analysis, and thereby address the limitations of examining a relatively small number of cases. The pipeline specifically included feature normalization to ensure fair comparisons, bootstrap-based consensus clustering to obtain stable uncorrelated features, diverse feature selection methods for informative feature identification, and time-to-event survival algorithms to explore associations with event outcomes. Based on a CV estimation of the performance, a PFS prediction was conducted by directly applying the pipeline to the cohort of 18 HGG patients with 2 PET scans, referred to as the default strategy.

For the default strategy, delta radiomics, whether assessing absolute or relative changes, outperformed delta conventional features and single-time-point radiomics features (Table 1). Similar results were found for the weighted delta radiomics (Supplementary Table S3). These findings suggest that delta radiomics may capture more pertinent information for the PFS prediction task, irrespective of the specific time interval between the two collected PET scans. In general, models from the default strategy yielded superior performance when evaluated with a single selected feature. This may be attributed to the small number of longitudinal samples, as fewer features made it easier to identify meaningful patterns and extract relevant information.

To address the limited number of samples with 2 PET scans, several robustness criteria were assessed to validate the default strategy. First of all, we developed the fine-tuned strategy applied to a larger cohort of 35 patients with a single PET scan, and subsequently, the information obtained was used to evaluate delta features within the smaller cohort of 18 patients. Interestingly, the fine-tuned strategy also exhibited a similar trend than the default one, highlighting the efficiency of delta radiomics, particularly when integrating the SVM algorithm with the RFE method for feature selection (Table 2). In both strategies, the delta radiomics extracted from dynamic TTP parametric images, along with the same second-order radiomics family (GLCM), were identified as the most informative features, reinforcing the prognostic value of dynamic analysis^33^. Notably, the GLCM Information Correlation 2 feature played a significant role in the default strategy, while the GLCM Cluster Prominence feature was crucial for the fine-tuned strategy (Fig. 3).

Dealing with the common challenge of limited sample size in rare cancer cohorts, it is essential to implement a robust pipeline, as attempted in this study. Additional criteria for ensuring the robustness of the default strategy included the following considerations. The methods used for computing changes in radiomics features did not show a notable impact on the outcomes, suggesting that the model reliably extracted pertinent information from the input features. Likewise, most radiomics models demonstrated superior performance compared to their conventional pairs, highlighting the potential efficacy of the default strategy (Fig. 2). In addition, integrating bootstrap-based consensus clustering into the dimensionality reduction step of the pipeline resulted in an average ARI value of 0.59 across CV folds for the best model within the default strategy, and subsequent evaluation of medoid features demonstrated consistency (Fig. 4). Interestingly, the GLCM Information Correlation 2 attribute emerged as the highest rank feature across all 16 model combinations (Fig. 5).

Some studies have explored the effectiveness of the delta SUV_max_ feature in predicting PFS and overall survival in diffuse large B-cell lymphoma^55–57^. However, in the present study, the analysis of the delta feature demonstrated that, for both strategies, delta radiomics performed better than delta TBR_max_, the normalized version of SUV_max_ using the mean of the brain (Tables 1 and 2, p < 0.001 when comparing delta-absolute of radiomics with TBR_max_ for both strategies, and p = 0.003 and p < 0.001 when comparing delta-relative of radiomics with TBR_max_ for default and fine-tuned strategies respectively) and delta TBR_mean_, the normalized version of SUV_mean_ using the mean of the brain (Tables 1 and 2, p < 0.001 when comparing the results of delta-absolute/relative radiomics features with their corresponding pairs of TBR_mean_ feature for both default and fine-tuned strategies) showcasing the superior performance of delta radiomics. This finding emphasizes the importance of further investigation within established guidelines such as PERCIST^58^. It is crucial to note that to comprehensively understand the implications of radiomics features, conducting additional evaluations over a larger cohort with more diverse samples is essential. Several studies have investigated deep learning and multitask learning approaches for analyzing time-to-event survival data^39,59–61^. While these algorithms demonstrate efficiency at capturing non-linear temporal patterns and managing temporal relationships across multiple time points, they also introduce complexity and require a larger sample size for generalization. These methods could nevertheless be a matter of future research. Conversely, working explicitly with delta radiomics provides several advantages, including reducing feature dimensionality and directly capturing temporal changes for enhanced interpretability and computational efficiency, which is adapted to the small number of samples. The proposed pipeline in this work can effectively be applied to explore the potential of radiomics changes over time in other rare patient cohorts.

The current study has several limitations. Firstly, as a retrospective study, the data was collected from a preselected patient population, thereby restricting patient inclusion. Given that our study was a single-center analysis, it would be useful to consider including samples from other centers or collecting data from new patients in our center for external validation to provide further verification in terms of the robustness and generalizability of the models developed. Admittedly, due to the retrospective nature of our study and the unavailability of raw data, we did not perform the repeatability of radiomics features in the current analysis. A feature repeatability assessment is, nevertheless worth considering in future studies to ensure reliable performance of the generated radiomics models^22,23^. Furthermore, although the study involved a limited number of patients who underwent 2 PET scans, the sample size corresponds to the typical range of longitudinal case numbers observed in rare diseases such as glioma^35–37^**,** and therefore reflects real life conditions. It is important to highlight that the primary objective of this study was to investigate the potential additive value of radiomics changes over time for predicting PFS in a small cohort of rare cancer patients. To address this, we implemented a robust pipeline and employed two distinct strategies, both of which yielded similar results, confirming the efficacy of the default strategy for delta radiomics.

## Conclusion

This study highlights the considerable potential of delta radiomics analysis when applied to a relatively small cohort of patients with rare HGG disease, and demonstrates its superior performance compared to both delta conventional features and single-point radiomics features. The proposed pipeline, assessed through robustness criteria and adapted to small case numbers, needs to be evaluated in other cancer indications to confirm the effectiveness of delta radiomics. Such analyses may lead to the development of novel prognostic biomarkers for patients with rare cancers.

### Supplementary Information

Supplementary Information.

## Data Availability

Available in supplementary information files.
